# Airway Management and Anesthesia for Tracheal Resection in a 68-Year-Old: 3 Airways for the Price of 1

**DOI:** 10.1155/2021/5548105

**Published:** 2021-12-27

**Authors:** Klint J. Smart, Iwan P. Sofjan

**Affiliations:** Department of Anesthesiology, Westchester Medical Center, 100 Woods Road, Valhalla 10595, NY, USA

## Abstract

Subglottic tracheal stenosis can occur after prolonged intubation or tracheostomy. This stenosis can become severe and causes symptoms refractory to endoscopic interventions that require tracheal resection. This surgery presents unique anesthetic issues due to the airway anatomy, physiology, and shared airway management with the surgical team. We present the case of a 68-year-old patient who underwent cervical tracheal resection and reconstruction due to persistent symptoms despite balloon dilation and medical management with oxygen and heliox. Our anesthesia management involved several techniques that allowed the safe completion of this procedure. Firstly, we started the airway management with a combined size 4 Ambu® AuraStraight™ (Denmark) supraglottic airway device and flexible bronchoscopy to allow localization of the stenosis and dilation before endotracheal tube (ETT) placement. The conventional approach for this endoscopic evaluation phase is to use rigid bronchoscopy. Secondly, we used prior CT images to help guide our ETT tube size selection. Thirdly, we used total intravenous anesthesia during most of the procedure because of the intermittent apnea necessary to complete the tracheal resection. Lastly, extubation had to be done very carefully to minimize excessive patient neck movement and avoid any reintubation. Both could lead to a catastrophe with the newly reconstructed trachea.

## 1. Introduction

Acquired tracheal stenosis is a late complication of prolonged endotracheal intubation (>7 days) and typically takes weeks to months to develop [1]. The signs and symptoms of upper airway obstruction such as stridor, dyspnea, and decreased exercise tolerance are associated with the extent of reduction of the intraluminal diameter [2]. This obstruction compromises effective airway oxygenation and ventilation. Surgical resection is considered the gold standard therapy for most patients with tracheal tumors or tracheal stenosis [3]. However, this surgery is difficult and carries a mortality rate of 3 percent when performed in experienced centers and 7–11 percent otherwise [2]. Management of these patients involves a multidisciplinary approach as well as close communication with the surgical team during tracheal excision and reanastomosis, proper extubation technique, and adequate postoperative care.

## 2. Case Description

**Figure 1:**
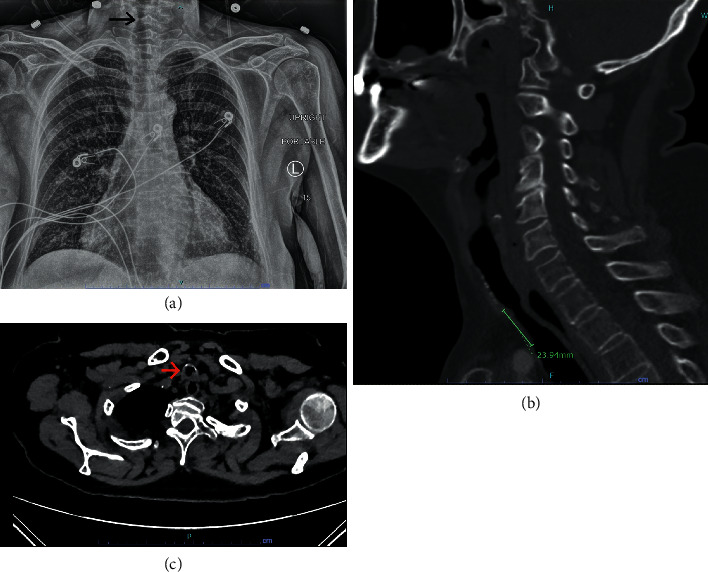
(a) Chest radiograph showing the stenosis in the upper third of the trachea; (b) sagittal section of the trachea showing the length of the stenotic segment; (c) axial cut of the narrowest segment of the trachea.

A 68-year-old female patient with tracheal stenosis presented for flexible bronchoscopy, tracheal stenosis resection, and reconstruction. Her past medical history was significant for chronic obstructive pulmonary disease, congestive heart failure, cardiomyopathy, atrial fibrillation, and coronary artery disease. Four months before surgery, she was hospitalized for acute decompensated heart failure, atrial fibrillation with rapid ventricular rate, and respiratory failure and was intubated for eighteen days in the intensive care unit (ICU). At that time, the patient was in severe cardiogenic shock from ischemic cardiomyopathy requiring an intraaortic balloon pump (IABP) placement for a few days. The patient underwent percutaneous coronary intervention with right coronary artery stenting and was medically managed then discharged home. She was readmitted 3 months after discharge for dyspnea, stridor, and decreased exercise tolerance. A computerized tomography (CT) scan of her neck showed short segmental narrowing of her trachea with the narrowest segment measuring 3 mm (Figure 1). Balloon dilatation was performed twice which only provided temporary relief of her symptoms. Medical management also included head of the bed elevation, supplemental humidified oxygen, and heliox therapy.

Preoperative transthoracic echocardiogram showed mild concentric left ventricular (LV) hypertrophy but normal LV and right ventricular function. Her left ventricular function significantly improved from her last admission. Chest radiography showed emphysema, old granulomatous disease, and cardiomegaly. Preoperative laboratory tests were unremarkable. On the day of surgery, physical examination was significant for moderate inspiratory stridor; however, her oxygen saturation was 96 percent on room air.

In the operating room, standard American Society Anesthesiology monitors were placed, along with a bispectral index monitor, 2 intravenous lines (18 gauge and a preexisting 22 gauge), and a right radial arterial catheter (20 gauge). We chose a size 4 Ambu® AuraStraight™ (Denmark) supraglottic airway (SGA) device with spontaneous ventilation as the initial airway after a discussion with our surgical team, which consisted of an interventional pulmonologist, a thoracic surgeon, and an otolaryngologist. Our interventional pulmonologist is well-versed in doing diagnostic and therapeutic airway management with the combined SGA and flexible bronchoscopy technique. In this case, the main purpose of the SGA was to allow visual localization and marking of the tracheal stenosis, which helped the surgeons plan their surgical approach. This process likely would have been more difficult with an endotracheal tube (ETT) because of the proximal stenosis location. Our interventional pulmonologist also planned to dilate the stenosis before switching to an ETT to reduce the risk of tissue injury. Our patient also had no absolute contraindication for the temporary SGA placement.

**Figure 2:**
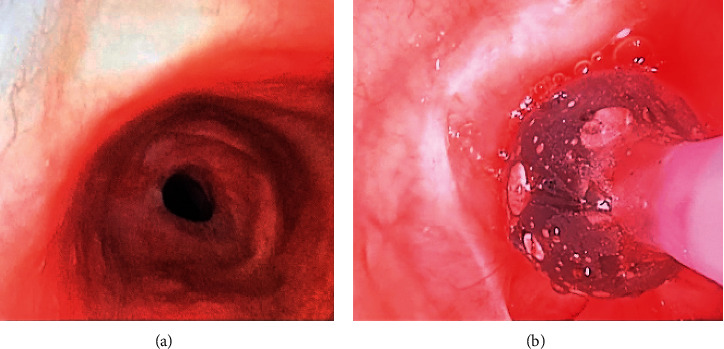
(a) Bronchoscopic view of the tracheal stenosis; (b) balloon dilation before initial endotracheal tube placement.

We opted for anesthesia induction with lidocaine (80 mg) and propofol (100 mg), which was utilized successfully on her last diagnostic bronchoscopy procedure under SGA and flexible bronchoscopy. The SGA was successfully placed, and the patient ventilated spontaneously under sevoflurane. Her vocal cords were sprayed with lidocaine to minimize laryngospasm during bronchoscopy in the absence of muscle relaxation. The stenotic area was identified 3-4 rings from the cricoid cartilage (Figure 2(a)) and then dilated with a balloon (Figure 2(b)). The scope was removed, anesthesia was deepened with inhaled sevoflurane, and the patient was subsequently intubated with a 6 mm internal diameter neural integrity monitor (NIM) ETT under video laryngoscopy to ensure proper positioning. Our otolaryngologist requested the NIM ETT for recurrent laryngeal nerve monitoring during dissection and surgical access of the trachea. Of note, the use of NIM ETT in tracheal resection has been done in several studies, which showed no absolute benefit if a proper surgical technique is performed [4, 5].

The position of the cuff of the tube was visualized distal to the stenotic region via bronchoscopy. From that point, total intravenous anesthesia (TIVA) was done with propofol (40–70 mcg/kg/min) and remifentanil (0.06–0.08 mcg/kg/min) infusions. The patient was placed on volume-controlled mechanical ventilation, and the fraction of inspired oxygen during the case was maintained at <0.3 whenever appropriate for airway fire precaution.

**Figure 3:**
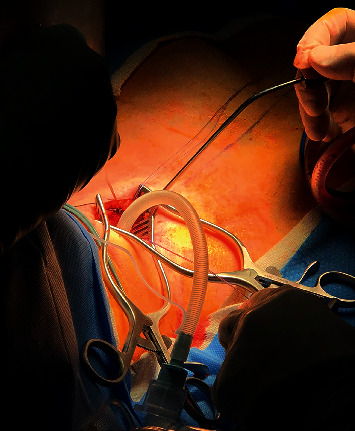
Subtracheal stenosis endotracheal ventilation.

The anterior surface trachea was exposed via surgical dissection. The exact location of the stenosis was then identified by combining bronchoscopy and needle localization in the surgical field. The anterior and posterior portions of the lower end of the stricture were divided. The NIM tube was withdrawn transorally into the subglottic area, and the distal trachea was intubated across the surgical field with a 6 mm internal diameter armored ETT (Figure 3), which is more pliable than a regular ETT. From that point, the distal trachea was cross-ventilated with intermittent periods of apnea and tube removal to allow for suture placement. Once a total of three stenotic tracheal rings were excised, sutures were placed on the proximal and distal ends of the trachea. The armored ETT was then removed from the distal trachea, and the NIM ETT was advanced transorally distal to the tracheal anastomosis to avoid injury to the coaptation line when reinflated. Ventilation was resumed and the anterior segment of the tracheal was repaired. At this stage, a bronchoscopic examination was done to assess the adequacy of the surgical anastomosis. The NIM ETT was then withdrawn, and the tracheal anastomosis appeared to be well coapted. Bag mask ventilation was carefully performed with 30 cmH_2_0, and no air leak was noted into the neck wound.

A guardian chin stitch was placed to limit neck extension before extubation. Nausea and vomiting prophylaxis was employed with ondansetron and TIVA. The patient was extubated awake while on a titrated remifentanil infusion to help minimize bucking and retching. She did not demonstrate any signs of respiratory distress, hemoptysis, or stridor, and supplement oxygen was administered with 2 liters per minute of oxygen through a nasal cannula. The remifentanil infusion was discontinued, and then, intravenous hydromorphone was carefully titrated for pain control. The patient was transferred to the cardiothoracic ICU as planned for close monitoring. There were no complications during the postoperative period course, the guardian chin stitch was removed on postoperative day 6, and the patient was discharged home on postoperative day 9.

## 3. Discussion

Frequent or prolonged intubation can lead to subglottic stenosis, which we speculate may become more common with our current prevalence of intubations due to coronavirus disease-2019 (COVID-19). Symptoms can range from mild to severe. In our patient, the symptoms included increased work of breathing, stridor, and exercise intolerance. Tracheal procedures such as mechanical dilatation, cryoablation, stenting, and laser-assisted dilatation all provide temporary relief in patients with mild to moderate symptoms [3, 6]. Severe or refractory symptoms require tracheal resection, which can significantly improve the quality of life and is associated with a >90 percent success rate [6]. Due to the need for specialized equipment, a shared airway with the surgeon, and abnormal airway physiology and anatomy, these surgeries provide unique challenges for anesthesiologists.

Endoscopic evaluation phase of the tracheal stenosis is typically done with rigid bronchoscopy or awake fiberoptic examination [2, 6]. In our case, we opted to employ general anesthesia with a combination of a SGA and flexible bronchoscopy to localize the tracheal stenosis and dilate the stenotic area before placement of an ETT. Other physicians that are highly skilled in airway management can be very helpful. In our case, the interventional pulmonologist was essential in helping determine the precise location of the lesion and performed the balloon dilatation, which permitted the passage of the ETT past the lesion without causing mucosal injury. Of note, measuring the tracheal diameter in the head and neck CT images helped us determine our ETT size selection.

During the procedure, the ETT cuff was inflated beyond the stenotic region because a circumferential incision is made at the lesion around the airway which could have damaged the cuff. During bronchoscopy evaluation, it is essential that an assortment of flexible bronchoscopes, balloon dilators, rigid bronchoscopes, and instruments to perform an emergent tracheostomy be readily available [4]. TIVA was the preferred anesthetic technique for this case because it ensured consistent anesthesia delivery during airway manipulation. TIVA also allowed avoidance of neuromuscular blockade, which was needed in this case because we utilized a NIM ETT to monitor the integrity of the recurrent laryngeal nerves during the extensive surgical dissection. However, if a proper surgical technique is performed, the incidence of recurrent laryngeal nerve injury is rare [4].

Due to the open trachea, airway fire is a concern and the fraction of inspired oxygen (FiO_2_) was kept <0.3 whenever possible. Intermittent interruptions in ventilations were also necessary to facilitate the surgical repair, which required close communication with the surgical team. An open technique with intermittent periods of apnea by removing the cross-field endotracheal tube is preferred by surgeons because it allows for careful, precise placement of sutures and proper spacing to address any size discrepancies [7]. Before initially starting the cross-field ventilation and before all periods of apnea, the patient should be preoxygenated with >0.9 FiO_2_. Our patient had 2 episodes of desaturation when the distal ETT was temporarily removed for the placement of anastomotic sutures requiring reintubation and manual hand ventilation with 100 percent oxygen.

Anesthesiologists should also be familiar with the possible postoperative complications of tracheal resection. One serious complication is a tracheal anastomotic rupture, which can occur with severe neck extension or traumatic reintubation with a rigid stylet. Our patient's chin was stitched before extubation to minimize neck extension. Careful extubation and close postoperative monitoring, ideally in the ICU, are crucial. We also explicitly communicated to the ICU and our intubation team to be very careful with reintubation, which ideally should include bronchoscopic guidance in placing the ETT to avoid injuring the new tracheal anastomotic line. Other immediate complications after tracheal resection include respiratory failure, vocal cord paralysis, and hemoptysis. Late complications include recurrent aspirations, infection, and hemoptysis.

In summary, airway management for tracheal resection is challenging for anesthesiologists. Incorrect clinical judgment during critical points of the surgery may result in devastating respiratory complications. Judicious extubation planning and technique are imperative to prevent compromise to tracheal anastomosis and respiratory failure.
